# The attrition, physical and insecticidal durability of two dual active ingredient nets (Interceptor® G2 and Royal Guard®) in Benin, West Africa: results from a durability study embedded in a cluster randomised controlled trial

**DOI:** 10.1186/s13071-024-06504-1

**Published:** 2024-10-07

**Authors:** Corine Ngufor, Josias Fagbohoun, Augustin Fongnikin, Juniace Ahoga, Thomas Syme, Idelphonse Ahogni, Manfred Accrombessi, Natacha Protopopoff, Jackie Cook, Edouard Dangbenon, Arthur Sovi, Marie Baes, Olivier Pigeon, Damien Todjinou, Renaud Govoetchan, Germain Gil Padonou, Martin Akogbeto

**Affiliations:** 1 London School of Hygiene and Tropical Medicine (LSHTM), London, WC1E 7HT UK; 2 Centre de Recherches Entomologiques de Cotonou (CREC), Cotonou, Benin; 3 Panafrican Malaria Vector Research Consortium (PAMVERC), Cotonou, Benin; 4 Centre Wallon de Recherches Agronomiques (CRA-W), Gembloux, Belgium

**Keywords:** Durability, Attrition, Survivorship, Fabric integrity, Bioefficacy, Insecticide-treated net, Pyriproxyfen, Chlorfenapyr, Alpha-cypermethrin, Dual active ingredients, Interceptor®, Interceptor® G2, Royal Guard®

## Abstract

**Background:**

Studies evaluating the attrition, physical and insecticidal durability of dual active ingredient (AI) insecticide-treated nets (ITNs) are essential for making programmatic decisions regarding their deployment. We performed a prospective study embedded in a cluster randomised controlled trial (cRCT) to evaluate the attrition, fabric integrity and insecticidal durability of Interceptor® G2 (alpha-cypermethrin-chlorfenapyr) and Royal Guard® (alpha-cypermethrin–pyriproxyfen), compared to Interceptor® (alpha-cypermethrin) in Benin.

**Methods:**

A total of 2428 study nets in 1093 randomly selected households in five clusters per arm of the cRCT were monitored for ITN attrition and fabric integrity every 6–12 months post-distribution. Householders were further surveyed to investigate non-study net use and their preference for ITN fabric types used in the study nets. A second cohort of 120 nets per ITN type were withdrawn every 12 months and assessed for chemical content and insecticidal activity in laboratory bioassays. Alpha-cypermethrin bioefficacy was investigated using the susceptible *Anopheles gambiae* Kisumu strain, and chlorfenapyr and pyriproxyfen bioefficacy were investigated using the pyrethroid-resistant *Anopheles coluzzii* Akron strain. Net pieces were tested in WHO cone bioassays and tunnel tests for alpha-cypermethrin and in tunnel tests for chlorfenapyr; pyriproxyfen activity was assessed in cone bioassays as the reduction in fertility of blood-fed survivors using ovary dissection. Bioefficacy was expressed as the proportion of ITNs passing predetermined WHO criteria, namely knock-down ≥ 95% or 24/72 h mortality ≥ 80% or reduction in fertility ≥ 50%.

**Results:**

Overall ITN survivorship was 52% at 24 months and fell to 15% at 36 months. Median ITN survival time was lower with Royal Guard® relative to Interceptor® [1.6 vs 2.3 years; hazard ratio (HR) 1.49, 95% confidence interval (CI) 1.36–1.66; *p* < 0.001] and Interceptor® G2 (1.6 vs 2.1 years; HR 1.33, 95% CI 1.20–1.47; *p* < 0.001). Householders overwhelmingly preferred polyester nets over polyethylene nets (96%), and more Royal Guard® nets were replaced with spare polyester nets from previous campaigns. All Royal Guard® nets passed efficacy criteria for alpha-cypermethrin at all time points (100%) while ITN pass rates after 24 months had fallen to < 40% for pyriproxyfen and chlorfenapyr. The chemical content analysis showed a higher loss rate of the non-pyrethroid insecticides relative to the pyrethroids in each dual ingredient AI ITN; 74% vs 47% for Royal Guard® and 85% vs 63% for Interceptor® G2 at 36 months.

**Conclusions:**

The median ITN survival time for Interceptor® G2 (2.1 years) and Royal Guard® (1.6 years) in Benin is substantially lower than 3 years. Royal Guard® nets were discarded more quickly by householders, partly due to their low preference for polyethylene nets. The insecticidal activity of the non-pyrethroid insecticides in both dual AI ITNs was short-lived compared to alpha-cypermethrin. The results corroborate the findings from the cRCT conducted in Benin.

**Graphical Abstract:**

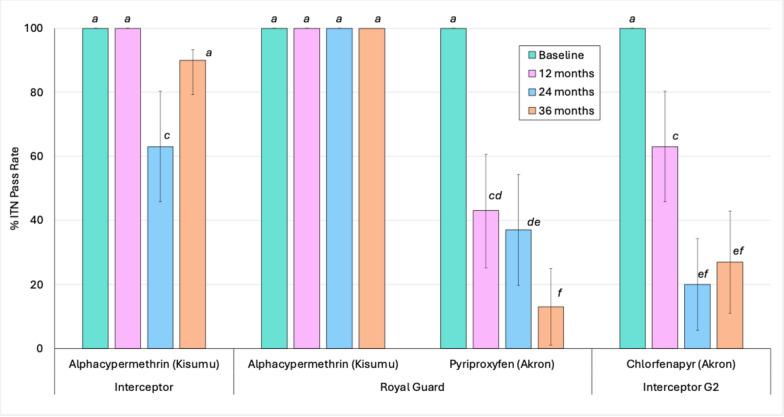

**Supplementary Information:**

The online version contains supplementary material available at 10.1186/s13071-024-06504-1.

## Background

The large-scale deployment of insecticide-treated nets (ITNs) via tri-annual mass campaigns is the main malaria prevention and control strategy deployed in most endemic countries in sub-Saharan Africa (SSA). While ITNs have played a major role in reducing the burden of malaria globally [1], their impact is threatened by their poor retention and use by householders, poor durability of the fabric and the insecticide applied to them and the development of vector resistance to pyrethroids, which are the main type of insecticides used on ITNs [2]. To optimise the impact of ITNs for malaria control, high levels of coverage of the target communities must be achieved, householders must retain and use the ITNs and the nets should maintain their efficacy against mosquito vectors until the next campaign cycle [3]. However, several studies have shown that ITNs are discarded more quickly than the 3 years presumed by country policies, with most malaria endemic African countries showing a median ITN retention time of < 2 years [4]. ITN retention rates vary widely from one community to another, driven by several factors including the durability of the fabric, usage patterns and perceived efficacy by householders [5]. To help inform programmatic decision-making for procurements and replacement of worn-out nets, the WHO has established guidelines for investigating the durability of ITNs under operational conditions [6]. In these guidelines, ITNs are followed longitudinally after distribution to investigate their survivorship, fabric integrity and insecticidal activity using well-defined procedures.

Several studies have reported poor entomological performance of pyrethroid-only ITNs in areas where vectors are resistant to pyrethroids. Dual active ingredient (AI) nets treated with a mixture of a pyrethroid and a non-pyrethroid compound capable of providing enhanced control of pyrethroid-resistant vectors were recently developed to help mitigate this threat. Nets treated with pyrethroids and the pyrrole chlorfenapyr or the insect growth regulator pyriproxyfen were endorsed by the WHO in 2023 [7] after improved public health value over 2 years of use had been demonstrated in cluster randomised controlled trials (cRCT) across Africa [8, 9]. Based on the findings from these trials, the WHO issued a strong recommendation for the deployment of pyrethroid-chlorfenapyr ITNs and a conditional recommendation for the deployment of pyrethroid-pyriproxyfen nets instead of pyrethroid-only nets in areas of pyrethroid resistance [7]. Following this endorsement, the proportion of these nets deployed in SSA is projected to increase substantially with pyrethroid-chlorfenapyr nets potentially replacing pyrethroid-only nets [10]. As the uptake of dual AI nets increases, studies monitoring their durability should be performed in different settings to inform decision-making for their procurement and replacement by control programmes. WHO guidelines for assessing the attrition, fabric integrity, chemical content and insecticidal durability of ITNs under operational use [11, 12] were initially developed when pyrethroids were the only insecticides applied on ITNs and are being updated to cover new dual AI ITNs. This includes identifying standardised high-throughput methods and suitable mosquito strains for testing the insecticidal durability of non-pyrethroid insecticides, such as chlorfenapyr and pyriproxyfen, on nets. Unlike pyrethroids that knockdown and kill mosquitoes, chlorfenapyr is a pyrrole insecticide that acts on the insect mitochondria to slowly induce death [13] while pyriproxyfen is an insect growth regulator that sterilises adult female mosquitoes leading to a substantial reduction in offspring [14, 15].

In Benin, similar to most countries in Africa where malaria is endemic, many ITN brands show poor durability. Indeed, a recent statistical inference study reported a low median ITN retention rate of approximately 1.4 years for Benin, placing it among the bottom six of the 40 malaria-endemic African countries included in the study [4]. To investigate the durability of dual AI nets in Benin, we nested a prospective cohort study in a cRCT conducted in the Zou region of the country between 2020 and 2023, with the aim to compare the durability of each of two dual AI ITNs, namely a pyrethroid–chlorfenapyr ITN (Interceptor® G2) and a pyrethroid-pyriproxyfen ITN (Royal Guard®), to that of a pyrethroid-only ITN (Interceptor®). We marked two cohorts of nets at baseline in 10 clusters in each arm of the cRCT and surveyed them every 6–12 months to assess ITN attrition rate, fabric integrity, insecticidal efficacy and chemical content over 3 years of household use.

## Methods

### Study area

The ITN durability study was performed in the Cove, Zagnanado and Ouinhi Districts (COZO) of the Zou Department of central Benin (7°11′N, 1°59′E) between March 2020 and November 2023. The study was nested in a cRCT evaluating the efficacy of dual AI nets for the control of clinical malaria compared to that of a pyrethroid-only net [16, 17]. The study area encompassed 123 villages with approximately 54,000 households and a population of 220,000 inhabitants. The area has two rainy seasons (May–July and September–November) although malaria transmission occurs year-round. A baseline survey performed in 2019 prior to the cRCT showed a high malaria infection prevalence of 43.5% despite high ITN use (96%) [18]. The vector population consists of both *Anopheles gambiae* and *Anopheles coluzzii* and is characterised by a high intensity of resistance to pyrethroids and susceptibility to chlorfenapyr and pyriproxyfen [19]. The main economic activities carried out by the population are agriculture, fishing, hunting, trade and hospitality.

### Study arms

In the durability study, three different ITN types were evaluated in the three respective arms of the cRCT: two dual AI nets (Interceptor® G2 and Royal Guard®) were compared to a standard pyrethroid-only ITN (Interceptor®). The specifications of the three ITN types are as follows:

(i) Interceptor® G2 (BASF AGRO B.V., Arnhem, the Netherlands) is a WHO-prequalified 100-denier, polyester ITN coated with a mixture of alpha-cypermethrin and chlorfenapyr at target concentrations of 100 mg/m^2^ (± 25%) and 200 mg/m^2^ (± 25%), respectively. The bursting strength of the fabric is ≥ 405 kPa, and the mass per unit area is 40 g/m^2^ (± 10%) for 100 denier yarn.
(ii) Royal Guard® (Disease Control Technologies, LLC, Greer, SC, USA) is a WHO-prequalified 150-denier, polyethylene ITN incorporated with a mixture of alpha-cypermethrin and pyriproxyfen at target concentrations of 225 mg/m^2^ (± 25%) each. The bursting strength of the fabric is ≥ 450 kPa, and the mass per unit area is 45 g/m^2^ (± 10%) for 150 denier yarn.
(iii) Interceptor® (BASF AGRO B.V.), is a WHO-prequalified 100-denier, polyester ITN coated with alpha-cypermethrin at a target concentration of 200 mg/m^2^ (± 25%). The bursting strength of the fabric is ≥ 405 kPa, and the mass per unit area is 40 g/m^2^ (± 10%) for 100 denier yarn. Interceptor® is the control arm of the trial against which Interceptor G2 and Royal Guard® are compared.

### Study design

**Fig. 1:**
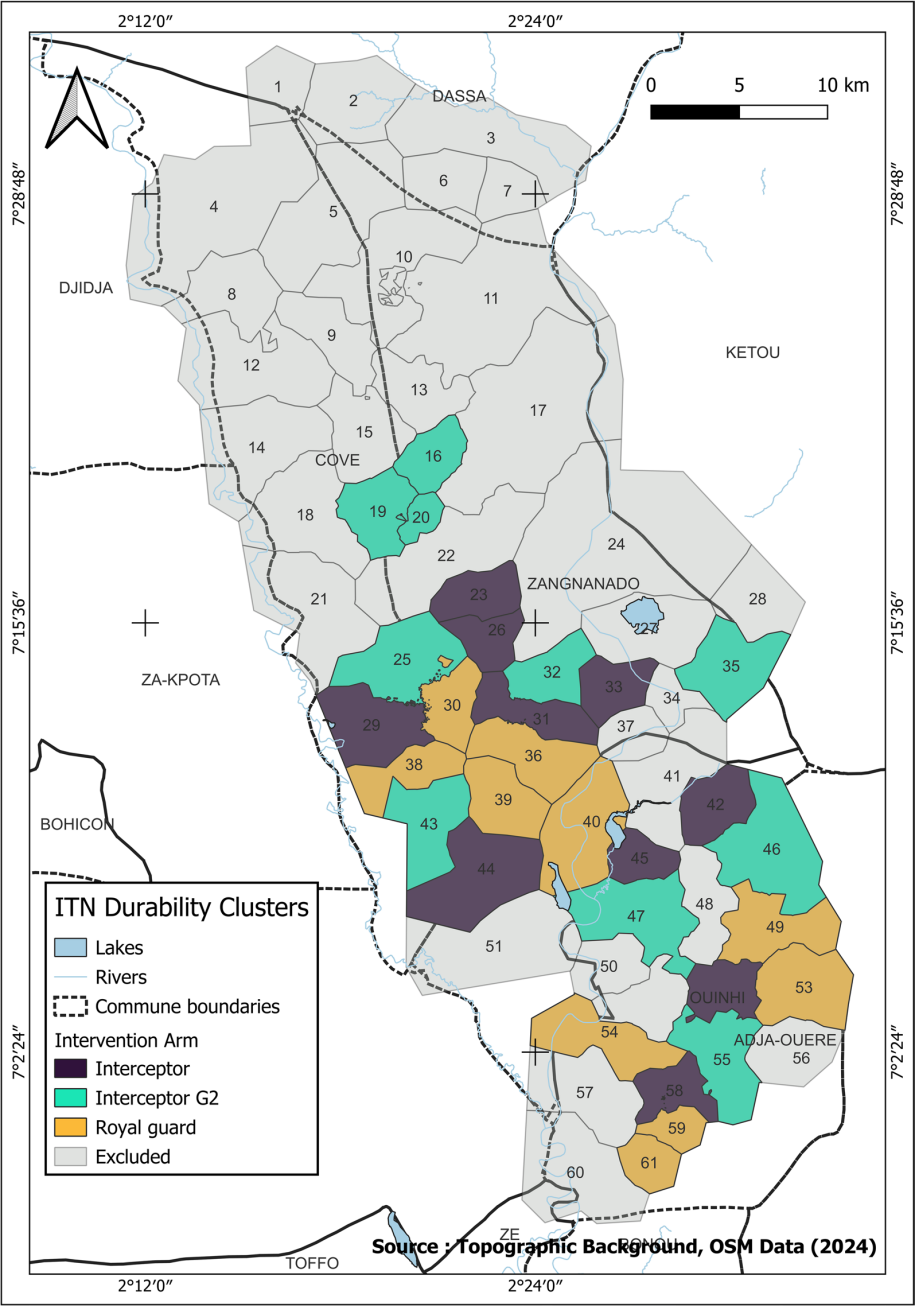
Map of study area of cluster randomised controlled trial showing ITN durability assessment clusters. Ten clusters were randomly selected from each study arm. ITN, Insecticide-treated net

The study design has been described in detail previously [17]. Participating households were blinded to the type of ITN they received, and field data collectors were blinded to the allocation of households to study arms. Of the 20 clusters of each study arm of the cRCT, 10 were randomly selected for the durability assessment (Fig. 1). Following ITN distribution, households were visited in these clusters to recruit and prospectively follow two separate cohorts of ITNs per arm (cohorts 1 and 2) every 6–12 months to monitor their survivorship, physical and insecticidal durability over 36 months. Cohort 1 consisted of a total of 2428 nets from 348 to 391 randomly selected households in five clusters per study arm. These nets were followed for ITN survivorship and fabric integrity at 6-, 12-, 24-, 30- and 36-months post ITN distribution. Cohort 2 consisted of 1860 nets per study arm from 593 households from all ten randomly selected clusters (approx. 60 households per cluster) per study arm that were withdrawn (and replaced with new nets) for assessment of insecticidal activity in laboratory bioassays and experimental hut trials, the content of each AI and for other studies. Cohort 1 and cohort 2 nets received separate identification numbers and were sampled from different households to prevent the risk of destructive sampling of nets intended for ITN survivorship studies (cohort 1).

### Household sensitisation and marking of study nets

The ITNs were recruited for the durability study in the first month after ITN distribution from selected clusters of each arm of the cRCT using the ITN distribution list of the trial. Field workers visited selected households for each cohort of ITNs to mark study nets available in these households. Using a wash-resistant marker, the field workers wrote a unique code on the study net to indicate the study arm, cluster ID, household ID and the cohort to which it belonged. During this visit, the study team explained the durability study to the householders in their local language and assisted them in hanging up their nets if needed. Only nets that were hung up and in use at the start of the trial were included in the durability study. The GPS coordinates and characteristics of each household included in the durability assessment were also recorded.

### ITN attrition and fabric integrity (cohort 1)

Households in cohort 1 were visited at 6, 12, 24 and 36 months post ITN distribution and nets were assessed for attrition and fabric integrity at each time point. Attrition was assessed by recording the physical presence/absence of each net that was recruited in the household. Where a net was not found, the householder was asked about the reason for the loss of the net (given away, sold, stolen, worn out, disposed of, among other reasons). Attrition was classified into three categories: (i) due to physical damage (wear and tear); (ii) due to the net being removed (given away, stolen, sold or used in another location); (iii) due to the net being used for other purposes (repurposed). Nets that had never been used were recorded and excluded from the analysis. At each time point, all available cohort 1 ITNs of each type were assessed for fabric integrity (hole index) and condition. A minimum of 250 nets were assessed and to ensure this number was reached, cohort 2 nets were also assessed for fabric integrity when the number of cohort 1 nets available was insufficient. The survey team inspected the nets outside the home in broad daylight to determine the hole index, using a portable frame over which the net was draped during the inspection. The nets were returned to the family immediately after the inspection.

The number of holes and hole sizes on each ITN panel was measured using hole assessment sheets and then classified into four sizes (size 1: 0.5–2 cm; size 2: 2–10 cm; size 3: 10–25 cm; size 4: > 25 cm). Physical integrity was measured as the proportionate hole index (pHI) which was calculated as pHI = (1 × number of size 1 holes) + (23 × number of size 2 holes) + (196 × number of size 3 holes) + (576 × number of size 4 holes). Nets were then categorised based on recommended cut-off points for pHI into “good” condition (pHI 0–64), “acceptable” condition (pHI 65–642) and “torn” (pHI 643+), as defined in WHO guidelines [12].

To determine the proportion of study nets that were in active use at each time point, we recorded the proportion of nets that were found hanging over sleeping places during each survey. Previous studies in Benin have indicated that householders usually have spare new ITNs from previous campaigns or routine distribution channels in their possession (unpublished data) which may affect the frequency at which the study nets are discarded. At 24 months, 362 participating households that had lost their study nets were further surveyed to identify the number, type and sources of non-study nets that they had replaced their study nets with. Householders were also administered a short questionnaire to determine which ITN fabric texture type (polyester vs polyethylene) they preferred and to provide the reasons for their stated preference. They were shown examples of the two ITN fabric types and allowed to touch them before providing their responses.

### ITN bioefficacy and chemical analysis (cohort 2)

For each ITN type a total of 120 nets belonging to cohort 2 were randomly selected and destructively sampled from households at 6-, 12-, 24- and 36-months post-distribution for laboratory and chemical analysis (Table 1). They were replaced with new nets of the same type at each sampling point. Thirty nets of each ITN type withdrawn at each time point were subjected to laboratory bioassays (WHO cone bioassays and tunnel tests where necessary) to monitor entomological efficacy against mosquito vectors and to chemical analysis to monitor changes in alpha-cypermethrin, chlorfenapyr and pyriproxyfen content. Four to five net pieces measuring 30 × 30 cm cut from each ITN at positions described in WHO guidelines (Figure S1), were tested in laboratory bioassays. A similar number of net pieces were obtained from adjacent positions to those for laboratory bioassays and preserved at 4 °C for chemical analysis.

**Table 1:** Number of Interceptor® G2, Royal Guard® and Interceptor® insecticide-treated nets tested for bioefficacy and chemical content

Months post ITN distribution (*n*)	Number of ITNs tested in lab bioassays	Number of pieces sampled per ITN	Total number of pieces for bioassays	Number of pieces for chemical analysis^a^
0	30	5	150	150
12	30	4	120	120
24	30	4	120	120
36	30	4	200	120
Total	120		590	510

*ITN* Insecticide-treated nets

^a^Net pieces preserved for chemical analysis were obtained from adjacent positions on the same ITNs cut for bioassays

#### Mosquito strains for laboratory bioassays

Cone bioassays and tunnel tests were performed to monitor the bioefficacy of each AI in each ITN brand using laboratory-reared susceptible and pyrethroid-resistant strains of *Anopheles gambiae* sensu lato (*An. gambiae* s.l.). All strains were maintained at the Centre de Recherche Entomologique de Cotonou/London School of Hygiene & Tropical Medicine (CREC/LSHTM) insectary in Cotonou, Benin. The characteristics of each strain are as follows:

- *An. gambiae* s.l. Kisumu is an insecticide-susceptible reference strain originating from the Kisumu area in Kenya. This strain was colonised at the CREC/LSHTM insectary.
- *An. coluzzii* Akron is a pyrethroid- and carbamate-resistant strain originating from Akron (9°19′N, 2°18′E), South Benin. It is maintained at the CREC/LSHTM insectary. Resistance is mediated by target site mutations (L1014F *kdr* and *Ace-1R*) and overexpressed cytochrome P450 enzymes [20].

WHO susceptibility bioassays were performed during each round of bioassays to confirm the susceptibility status of each mosquito strain to the insecticides used on the nets. At each time point, approximately 100 mosquitoes of each strain were exposed in batches of 25 in WHO bottle bioassays treated with alpha-cypermethrin (0.05%), chlorfenapyr (100 μg) or pyriproxyfen (100 μg). For the alpha-cypermethrin and chlorfenapyr bioassays, unfed 2- to 5-day-old mosquitoes were exposed for 1 h, and mortality was recorded after 24 h for alpha-cypemethrin and 72 h after exposure for chlorfenapyr. For pyriproxyfen, blood-fed 5- to 8-day-old mosquitoes were exposed for 1 h in treated bottles, and survivors were dissected 3 days later to measure the proportional reduction in fertility relative to the control unexposed mosquitoes, as described previously [21]. The bioassays were performed at a temperature of 27 °C ± 2 °C and relative humidity of 75% ± 10%.

#### Laboratory bioassay methods

In accordance with the existing WHO guidelines [6, 12], ITN bioefficacy at each time point was expressed in terms of the proportion of ITNs passing pre-determined efficacy criteria for each AI in cone bioassays or tunnel tests. The methods and strains used to assess the efficacy of each AI in each ITN type are summarized in Additional file 1: Table S1. All bioassays were performed at 27 ± 2 °C and 75% ± 10% relative humidity.

##### Bioefficacy of alpha-cypermethrin in the Interceptor® and Royal Guard® ITNs

 Following WHO guidelines for testing the durability of the bioefficacy of pyrethroid-treated ITNs, the efficacy of alpha-cypermethrin in the Interceptor® and Royal Guard® ITNs was monitored in 3-min cone bioassays using unfed 3- to 5-day-old mosquitoes of the susceptible *An. gambiae* Kisumu strain. At each time point, 40–50 mosquitoes were exposed to each ITN in replicates of approximately five mosquitoes per cone and two cones per ITN piece. ITNs that failed to achieve WHO efficacy criteria in these cone bioassays (pooled knock-down ≥ 95% or pooled 24 h mortality ≥ 80%) were subjected to tunnel tests. For each ITN that failed in the cone bioassays, only one ITN piece (with cone bioassay mortality that was closest to the mean) was tested in tunnels. Approximately 100 unfed 5- to 8-day-old susceptible *An. gambiae* Kisumu mosquitoes were exposed to each ITN piece in the tunnel tests, and the efficacy of alpha-cypermethrin in the Interceptor® and Royal Guard® ITN pieces was measured in terms of mortality after 24 h (≥ 80%) or blood-feeding inhibition (≥ 90%).

##### Bioefficacy of pyriproxyfen in the Royal Guard® ITN

To monitor the bioefficacy of pyriproxyfen in the Royal Guard® ITN, blood-fed 5- to 8-day-old mosquitoes of the pyrethroid-resistant *An. coluzzii* Akron strain were exposed to this pesticide for 3 min in cone bioassays and assessed for the impact on ovary development using dissection. Dissections were performed 72 h after exposure under a microscope, and mosquitoes were classified as fertile or infertile following previously described standard operating procedures [21–23]. A total of 80–100 blood-fed mosquitoes were tested against each Royal Guard® ITN in replicates of five mosquitoes per cone and four cones per ITN piece. The efficacy was measured for each ITN in terms of the proportional reduction in fertility relative to control unexposed mosquitoes. A cut-off of ≥ 50% reduction in fertility relative to the untreated control nets was applied to determine pass rates of Royal Guard® nets for pyriproxyfen bioefficacy. Only tests for which at least 30% of unexposed mosquitoes were found fertile were considered to be valid.

##### Bioefficacy of chlorfenapyr in the Interceptor® G2 ITN

 The bioefficacy of chlorfenapyr in the Interceptor® G2 ITN was assessed in tunnel tests using the pyrethroid-resistant *An. coluzzii* Akron strain. To improve the efficiency of the tunnel test bioassays, only one ITN piece per whole Interceptor® G2 ITN was tested in tunnels. For each tunnel, a total of approximately 100 unfed 5- to 8-day-old pyrethroid resistant *An. coluzzii* Akron were exposed overnight. The efficacy of Interceptor® G2 in tunnels was measured in terms of mosquito mortality after 72 h (≥ 80%) or blood-feeding inhibition (≥ 90%).

#### Chemical analysis methods

Net pieces preserved for chemical analysis at 0, 12, 24 and 36 months (510 pieces per ITN type) were wrapped in aluminum foil and stored at 4 °C (± 2 °C), following which they were shipped to Centre Walloon de Recherches Agronomiques (CRA-W), Belgium, for detection of fabric weight and AI content. The methods used for AI extraction have been described elsewhere [14, 24]. Gas chromatography with flame ionisation detection (GC-FID) was used to determine the content of each AI. ITN pieces from the same net were pooled to provide a single chemical content reading per AI for each whole ITN per net type sampled at each time point.

### Data management

Household data collected during the census and ITN follow-up surveys were captured on electronic forms using smartphones installed with OpenDataKit (ODK) Collect. Entomological data were recorded on data entry forms and double-entered pre-designed Excel (Microsoft Corp., Redmond, WA, USA) databases. Throughout the study, electronic data were stored encrypted while paper forms were locked up in secured cabinets and were available only to study investigators and data management staff by passwords and keys. All personal data were anonymised using a unique identifier number for each participant and household to ensure confidentiality. At the end of the study, all electronic files and data entry forms were stored on the server and archive of the CREC-LSHTM GLP-certified facility, respectively, where they will remain for 10 years.

### Statistical analysis

Data from the surveys at 6, 12, 24, 30 and 36 months were used to calculate attrition, functional survival and median survival time of the ITNs. Attrition was estimated at each time point as the proportion of study nets that were not found in households either due to physical damage, removal or repurposing relative to the number of nets distributed as a baseline after removing nets that were lost to follow-up. Functional survival was calculated at each survey time point as the proportion of nets in serviceable condition after excluding nets that were given away, sold or stolen. Median survival was calculated as the linear extrapolation of the survival values from either the 12- or 24-month rounds (whichever was < 85% survival) and the 36 months round to the* y* = 50% line, using the following formula:

$$Tm=t1+\left(t2-t1\right)*(p1-50)/(p1-p2),$$

where tm is the median survival time, t1 and t2 are the first and second time points in years and p1 and p2 are the proportions surviving to the first and second time points, respectively in percentage. Confidence intervals (CIs) for this estimate were calculated by projecting the 95% CI from the survival estimates in the same way as described above. Hazard ratios (HRs) for the difference in functional survival and 95% CIs were predicted using Cox proportional regression models. A Chi-squared test was used to assess the proportion of nets of each ITN type passing the WHO criteria for alpha-cypermethrin, chlorfenapyr and pyriproxyfen bioefficacy based on the results of the combined cone and tunnel tests.

## Results

### ITN survivorship, attrition rate and fabric integrity

#### ITN survivorship

A total of 2428 ITNs were recruited at baseline, consisting of 792 Interceptor®, 820 Royal Guard® and 816 Interceptor® G2 ITNs. Table 2 provides details on the number of nets that were available at each time point and the ITN functional survival rate calculated as the proportion of nets that were found in serviceable condition. Functional survival declined across all ITN types over time, falling to < 20% at the 36-month follow-up. The fastest decline in functional survival was observed with the Royal Guard® ITN (38% at 24 months and 6% at 36 months). Median ITN survival time was highest with Interceptor® (2.3 years; 95% CI 2.2–2.4 years; *p* < 0.05; Table 3). The Royal Guard® ITN also showed a significantly lower median survival time relative to Interceptor® [95% CI 1.6 (1.4–1.8) years vs 2.3 (95% CI 2.2–2.4) years; HR 1.49, 95% CI 1.36–1.66; *p* < 0.001] and to Interceptor® G2 [1.6 (95% CI 1.4–1.8) years vs 2.1 (95% CI 2.0–2.2) years; HR 1.33, 95% CI 1.20–1.47; *p* < 0.001].

**Table 2:** Functional survivorship of insecticide-treated nets

Time point	Functional survivorship parameters	ITN type			All ITNs
		Interceptor®	Royal Guard®	Interceptor® G2	
	Number of ITNs marked at baseline	792	816	820	2428
6 months	ITNs available for assessment (*n*)	696	635	698	2029
	ITNs lost (all causes) (*n*)	96	181	122	399
	% Attrition (95% CI)	12.1 (9.7–14.4)	22.2 (19.0–25.1)	14.9 (12.2–17.3)	16.4 (14.8–17.9)
	ITNs in serviceable condition (*n*)	680	607	662	1948
	ITNs given away (*n*)	110	178	109	397
	% Functional survival (95% CI)	99.7 (98.9–99.9)	94.5 (92.5–96.2)	93.6 (91.6–95.3)	95.3 (94.9–96.7)
12 months	ITNs available for assessment (*n*)	587	465	573	1625
	ITNs lost (all causes) (*n*)	205	351	247	803
	% Attrition (95% CI)	25.9 (22.3–28.9)	43.0 (38.5–46.4)	30.1 (26.4–33.3)	33.1 (30.8–34.9)
	ITNs in serviceable condition (*n*)	553	428	515	1496
	ITNs given away	216	278	201	695
	% Functional survival (95% CI)	96 (94.1–97.5)	79.0 (75.3–82.3)	83.7 (80.6–86.6)	86.3 (84.6–87.9)
24 months	ITNs available for assessment (*n*)	420	276	359	1055
	ITNs lost (all causes) (*n*)	372	540	461	1373
	% Attrition (95% CI)	47.0 (42.2–50.5)	66.2 (60.6–69.4)	56.2 (51.1–59.6)	56.5 (53.6–58.5)
	ITNs in serviceable condition (*n*)	370	222	299	891
	ITNs given away (*n*)	216	238	271	725
	% Functional survival (95% CI)	64.2 (60.2–68.2)	38.1 (33.2–42.2)	54.9 (50.6–59.1)	52.3 (49.9–54.7)
30 months	ITNs available for assessment (*n*)	278	139	236	653
	ITNs lost (all causes) (*n*)	514	677	584	1775
	% Attrition (95% CI)	64.9 (59.3–68.2)	83.0 (76.7–85.6)	71.2 (65.4–74.3)	73.1 (69.7–74.9)
	ITNs in serviceable condition (*n*)	192	104	161	457
	ITNs given away (*n*)	395	236	433	1064
	% Functional survival (95% CI)	48.3 (43.3–53.4)	17.8 (14.8–21.1)	42 (37.0–47.2)	33.5 (31.0–36.1)
36 months	ITNs available (*n*)	154	60	165	165
	ITNs lost (all causes) (*n*)	638	756	655	2263
	% Attrition (95% CI)	80.6 (74.3–83.3)	92.6 (86.0–94.4)	79.9 (73.8–82.6)	93.2 (89.4–94.2)
	ITNs in serviceable condition (*n*)	135	45	131	131
	ITNs given away (*n*)	495	333	540	540
	% Functional survival (95% CI)	19.5 (16.6–22.7)	6.2 (4.6–8.2)	18.4 (15.7–21.5)	14.6 (13.2–16.2)

*CI* Confidence interval,* ITN* insecticide-treated net

**Table 3:** Median survival times

ITN type	Median survival time, years (95% CI)	Hazard ratio (95% CI)	*p* value
Interceptor®	2.3 (2.2–2.4)	1	N/A
Royal Guard®	1.6 (1.4–1.8)	1.49 (1.36–1.66)	*p* < 0.001
Interceptor® G2	2.1 (2.0–2.3)	1.13 (1.02–1.26	*p* = 0.027
All ITNs	2.1 (2.0–2.1)	N/A	N/A

*CI* Confidence interval,* N/A* not applicable, * ITN* insecticide-treated net

#### ITN fabric integrity and pHI of study nets

**Fig. 2:**
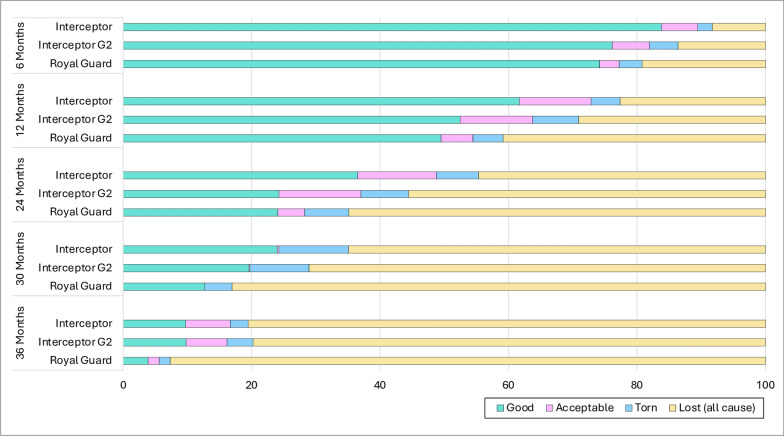
Proportionate hole index of the study nets at each survey time point

Figure 2 shows the pHI of nets that were found and assessed for fabric integrity at each time point. The proportion of nets that were found in good and acceptable condition relative to the numbers distributed at baseline had declined substantially over time and was < 40% at 24-months post distribution; at 36-months post distribution, < 20% of nets were found in good or acceptable conditions relative to the numbers distributed at baseline.

#### Reasons for ITN attrition

**Fig. 3:**
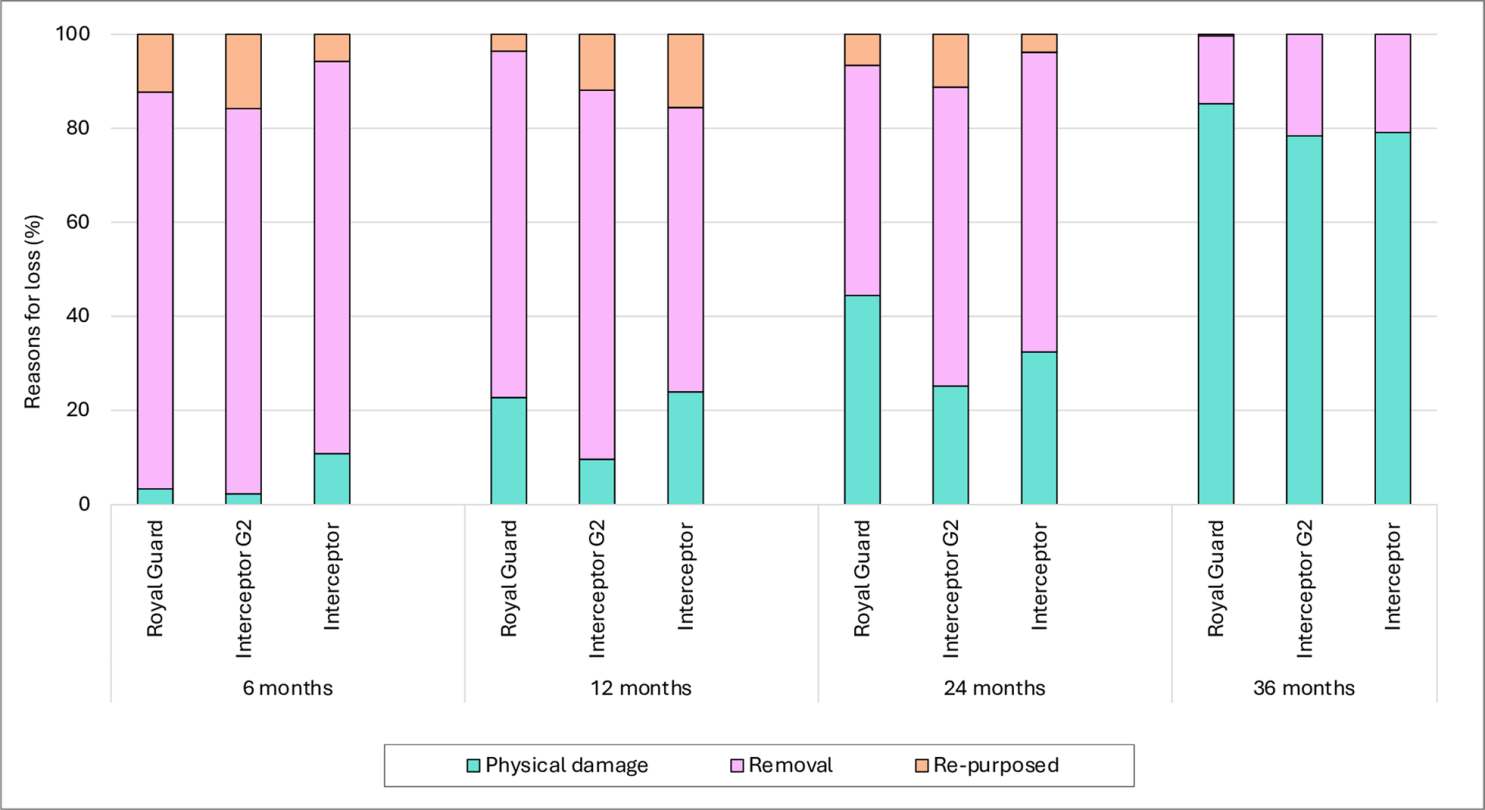
Reasons for attrition of study nets at each survey time point. Attrition was classified as being due to wear and tear (physical damage) (**a**), due to the net being given away, stolen, sold or used in another location (removal) (**b**) and due to the net being used for other purposes (repurposed) (**c**)

The most common reason for ITN attrition in the first 24 months of the study was that the nets were used elsewhere or given away to relatives (Fig. 3). The proportion of ITNs that were discarded due to physical damage was < 10% at 6 months, increasing to 25–45% at 24-months post distribution. By the 36-month survey, approximately  80% of ITNs had been discarded due to physical damage.

### Non-study nets in use and ITN preference survey

**Fig. 4:**
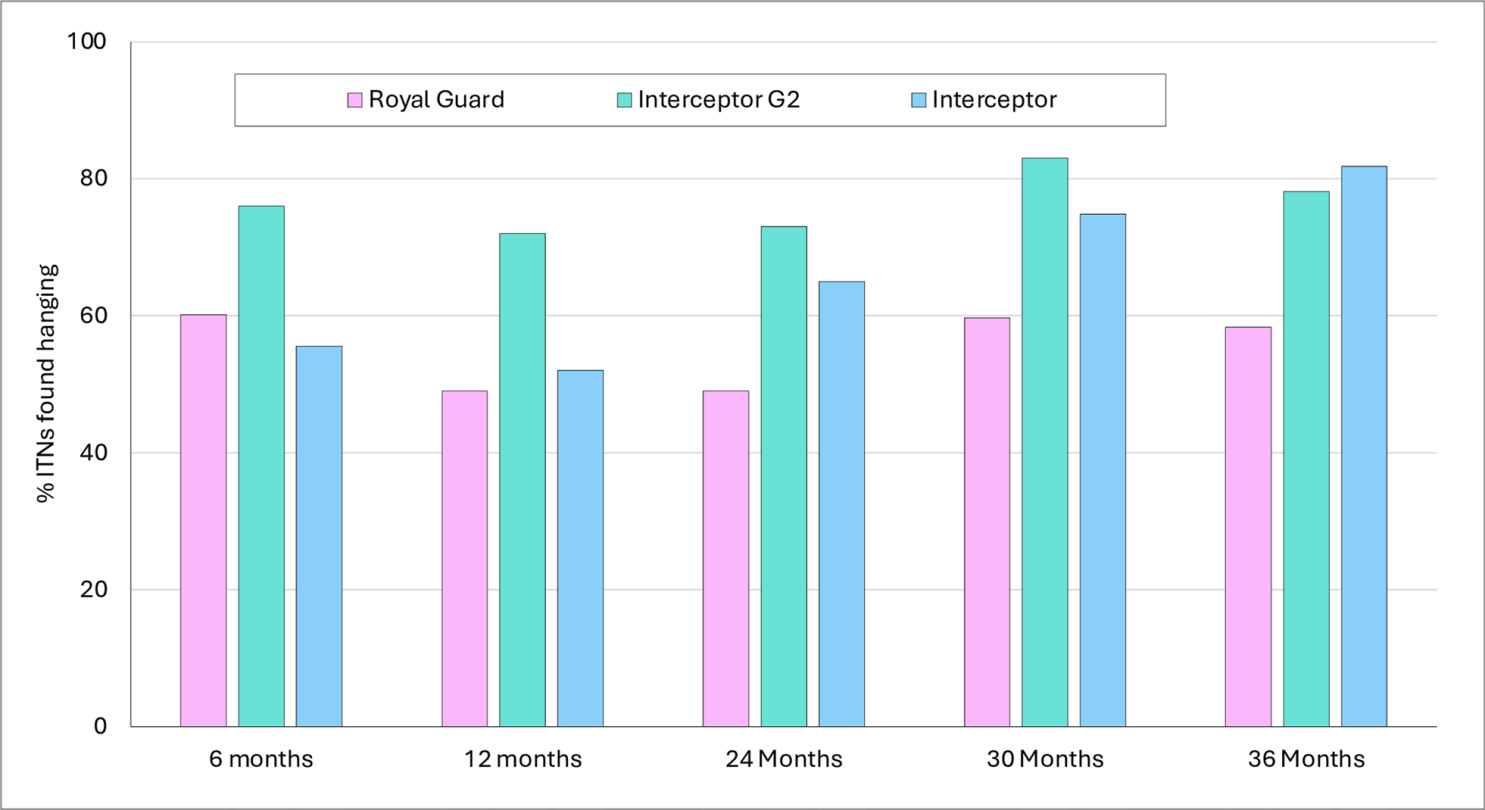
Proportion of study nets hanging over sleeping places at each survey time point. ITNs, Insecticide-treated nets

Approximately 50–80% of study nets that were found and assessed at each time point were hanging over sleeping places, indicating active use of the nets (Fig. 4). For Interceptor® G2, this proportion was > 70% at all time points; in comparison, for Interceptor®, the proportion of hanging nets was relatively lower at 6 and 12 months (50–60%) but increased substantially at later time points. For Royal Guard®, there were in general low hanging rates of 50–60% across all time points, indicating a lower use rate. Table 4 shows the number of non-study nets that were found hanging in households in each study arm that were surveyed at 24-months post-distribution; the aim of this assessment was to investigate the replacement of study nets with other ITN types. We found a total of 761 non-study nets in 362 surveyed houses, of which 75% were PermaNet® 2.0 nets (Vestergaard, Lausanne, Switzerland) that had been distributed in the previous ITN mass campaign. The highest proportion of non-study nets in use was found in households in the Royal Guard® arm (52%), indicating a higher replacement of Royal Guard® nets relative to Interceptor® G2 (14.3%) and Interceptor® (33.5%) nets. Of a total of 300 heads of households surveyed for ITN fabric texture preference, 96% indicated a preference for polyester net fabric over polyethylene net fabric. The polyester fabric texture was perceived to be softer, cooler and less abrasive on the skin compared to the polyethylene fabric texture. About 60% of householders believed that the polyethylene nets shrunk in size after washing compared to 4% of householders for polyester nets.

**Table 4:** Proportion of non-study nets in use per study arm

Measure	ITN type			All ITNs
	Royal Guard®	Interceptor G2®	Interceptor®	
Number of householders surveyed	138	110	114	362
Number of non-study nets in use	397	109	255	**761**
Percentage of non-study nets in use	52.2	14.3	33.5	100

* ITN* Insecticide-treated net

### Bioefficacy in laboratory bioassays

#### Susceptibility of test strains

**Fig. 5:**
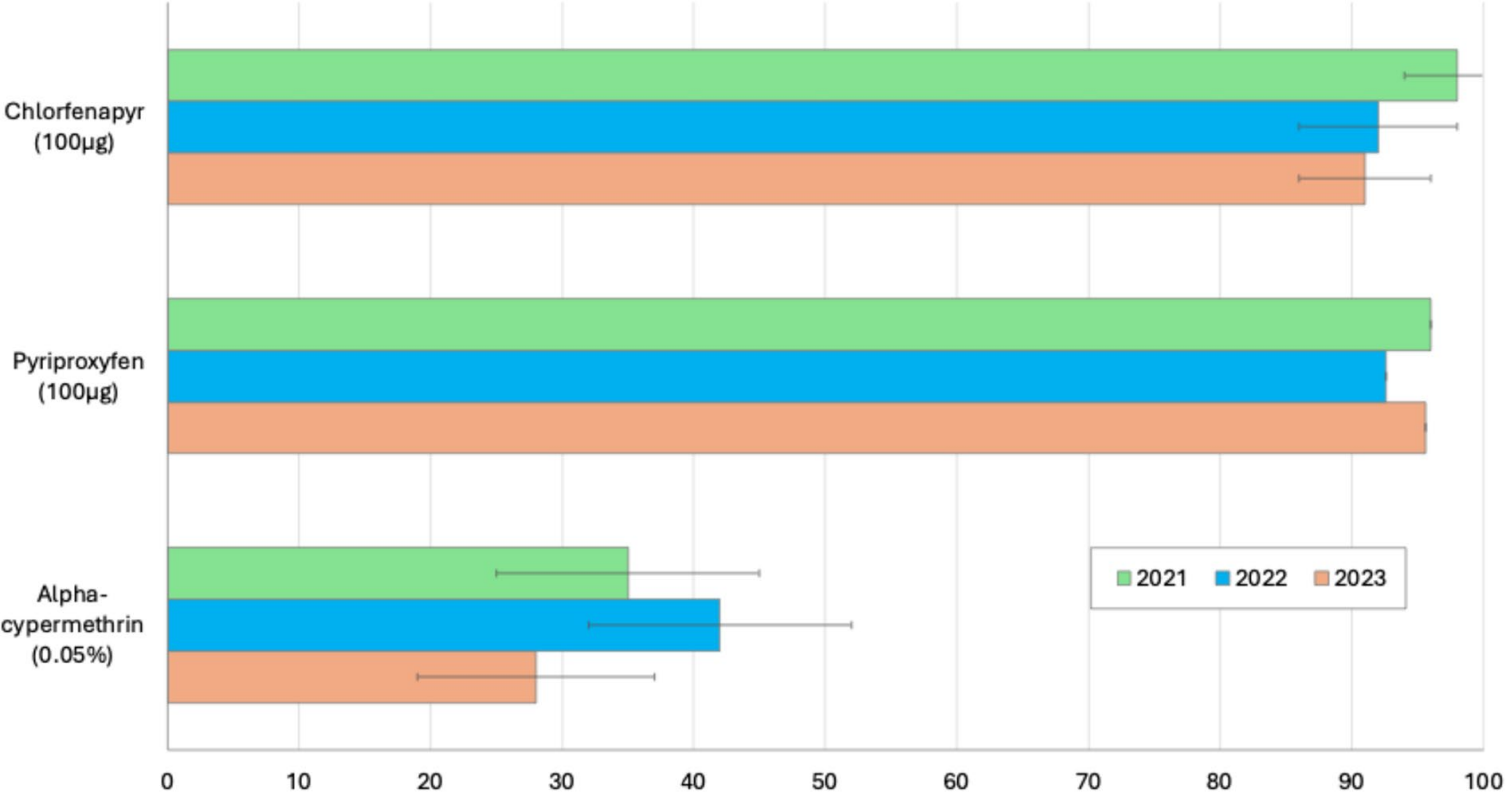
Susceptibility of pyrethroid-resistant *Anopheles coluzzii* Akron strain mosquitoes to insecticides applied on study nets. Approximately 100 mosquitoes were exposed to each insecticide at each time point

Figure 5 presents the annual results obtained in the susceptibility bioassays performed with the two mosquito strains (Kisumu and Akron) tested against the study nets in the laboratory bioassays. For the Akron strain, during each year of the study, mortality in the alpha-cypermethrin-treated tube tests remained < 50% while mortality in the chlorfenapyr-treated bottles remained > 90%; these results showed that the strain had maintained its resistance to pyrethroids and susceptibility to chlorfenapyr. The reduction in fertility of the Akron strain mosquitoes exposed to pyriproxyfen in bottle bioassays was also > 90% during each year of testing, showing the susceptibility of this strain to the insecticide throughout the study. Mortality in further bioassays performed with 5× and 10× the diagnostic dose of alpha-cypermethrin was < 95% at all time points, demonstrating that the Akron strain had a high intensity of pyrethroid resistance throughout the study. Susceptibility bioassays performed with the Kisumu strain also showed that the strain remained susceptible to all three insecticides through the course of the study.

#### Bioefficacy results with the Interceptor® ITN

A total of 7627 susceptible *An. gambiae* Kisumu strain mosquitoes were exposed to Interceptor® nets in 3-min cone bioassays at baseline and at 12, 24 and 36 months after household use. Overall mosquito mortality declined from 76.8% at baseline to 58.3% at 24 months and 44.4% at 36 months after household use (Table 5). Approximately one-half of the nets tested in cone bioassays failed to achieve WHO criteria in cone bioassays (knock-down ≥ 95% or 24 h mortality ≥ 80%) and were thus subsequently tested in tunnel tests. Mosquito mortality in the tunnel tests was 98–100% up to 12 months but declined to 53.7% at 24 months before increasing to 87.4% at 36 months. Overall blood-feeding inhibition in tunnels generally exceeded 90% except at 24 months when it was 79.7%. Mortality with control untreated ITN pieces did not exceed 5% at any time point. Blood-feeding inhibition in tunnel tests was > 75% at each time point. All 30 Interceptor® ITNs passed efficacy criteria in either the cone bioassays or tunnel tests at baseline and 12 months although the numbers which passed fell to 19 at 24 months and later increased to 27 at 36 months.

**Table 5:** Results with Interceptor® nets tested against susceptible *Anopheles gambiae* Kisumu strain mosquitoes in cone bioassays and tunnel tests at baseline and at 12-, 24- and 36-months post-distribution

Bioassays	Time points			
	Baseline	12 months	24 months	36 months
*Number of ITNs tested*	30	30	30	30
*Cone bioassay results*				
Number of ITN pieces tested	150	120	120	120
Number of ITN pieces exposed	1530	1233	2326	2538
Number of KD	1394	1187	1961	1963
Number of dead mosquitoes	1175	934	1355	1126
% KD (95% CI)	91.1 (86.3–92.5)	96.3 (90.8–97.4)	84.3 (80.6–85.8)	77.3 (73.9–78.9)
% Dead (95% CI)	76.8 (72.4–78.9)	75.8 (70.9–78.2)	58.3 (55.2–60.3)	44.4 (41.8–46.3)
*Tunnel test results with failed nets from cone bioassays*^*a*^				
Number of ITN pieces tested	14	6	15	16
Number of ITN pieces exposed	1589	543	1519	1602
Number of dead mosquitoes	1564	543	815	1400
Number of blood-fed mosquitoes	95	1	240	102
% Dead (95% CI)	98.4 (93.5–99.0)	100 (96–100)	53.7 (50.0–56.2)	87.4 (82.8–89.0)
% Blood-fed	6 (4.8–7.2)	0.2 (0–0.6)	15.8 (13.8–17.6)	6.4 (5.2–7.6)
% Blood-feeding inhibition	92.3	99.7	79.7	91.8
Number of ITNs passing WHO criteria	30/30	30/30	19/30	27/30

*CI* Confidence interval,* ITN* insecticide-treated net,* KD* knock-down

^a^Only nets that failed WHO criteria in the cone bioassays were tested in the tunnel bioassays

#### Bioefficacy results with Royal Guard® ITN

Results obtained in bioassays investigating the bioefficacy of the Royal Guard® ITN are presented in Table 6. To assess the bioefficacy of alpha-cypermethrin in the Royal Guard ITN, we exposed a total of 8363 susceptible *An. gambiae* Kisumu strain mosquitoes to Royal Guard® nets in 3-min cone bioassays at baseline and at 12-, 24- and 36-months post-distribution. Overall mosquito mortality was > 90% at all time points of testing. All ITNs passed WHO criteria for alpha-cypermethrin efficiency in the cone bioassays and, therefore, tunnel tests were not performed. A total of 1966 blood-fed pyrethroid-resistant *An. coluzzii* Akron strain mosquitoes that survived exposure to Royal Guard® nets in the 3-min cone bioassays at baseline and at 12, 24 and 36 months were dissected to investigate the bioefficacy of pyriproxyfen. The overall reduction in fertility rate was 99.3% at baseline but declined substantially to < 40% at 12 and 24 months and was 24.3% at 36 months. Mortality among mosquitoes exposed to the control untreated net pieces did not exceed 5% at each time point, and fertility rates among the control unexposed mosquitoes were > 50%. All 30 Royal Guard® ITNs passed the criteria for alpha-cypermethrin bioefficacy at all time points. For pyriproxyfen bioefficacy, the number of Royal Guard® ITNs that passed efficacy criteria (> 50% reduction in fertility relative to control) was 30 at baseline, decreasing substantially to 13 at 12 months and then to only four at 36 months.

**Table 6:** Results with Royal Guard® nets tested in cone bioassays against susceptible *Anopheles gambiae* Kisumu strain mosquitoes and blood-fed pyrethroid-resistant* Anopheles coluzzii* Akron strain mosquitoes at baseline, 12, 24 and 36 months post-distribution

Bioassays	Time points			
	Baseline	12 months	24 months	36 months
*Number of ITNs tested*	30	30	30	30
*Cone bioassays with An. gambiae Kisumu strain mosquitoes to investigate alpha-cypermethrin bioefficacy*				
Number of ITN pieces tested	150	120	120	120
Number of mosquitoes exposed	1491	2351	2326	2468
Number of KD	1491	2345	2324	2434
Number of dead mosquitoes	1491	2315	2182	2378
% KD (95% CI)	100.0 (96–100)	99.7 (95.7–99.9)	99.9 (95.8–100)	98.6 (94.7–99.1)
% Dead (95% CI)	100 (96–100)	98.5 (94.5–99.0)	93.8 (89.9–100)	96.4 (92.5–97.1)
Number of ITNs passing WHO criteria	30/30	30/30	30/30	30/30
*Cone bioassays with blood-fed An. coluzzii Akron strain mosquitoes to investigate pyriproxyfen bioefficacy*				
Number of ITN pieces tested	150	120	120	120
Number of mosquitoes exposed	2803	1152	2438	2480
Number of mosquitoes dissected	143	217	873	950
Number of mosquitoes fertile	1	138	551	707
Number of mosquitoes unfertile	142	79	322	243
% Fertile (95% CI)	0.7 (0.4–1.0)	63.6 (59.0–66.4)	63 (59.9–64.9)	74.4 (71.0–76.1)
% Reduction in fertility relative to control	99.3	35.3	36.0	24.3
Number of ITNs passing WHO criteria	30/30	13/30	11/30	4/30

*CI* Confidence interval,* ITN* insecticide-treated net,* KD* knock-down

#### Bioefficacy results with Interceptor® G2 ITN

A total of 13,092 pyrethroid-resistant *An. coluzzii* Akron strain mosquitoes were exposed to Interceptor® G2 nets in tunnel tests throughout the course of the study. Overall mosquito mortality was 95.7% at baseline and declined to < 80% at subsequent time points (Table 7). Blood-feeding inhibition did not exceed 90% at any time point and had declined to < 45% by 24 months and onwards. Mortality in tunnel tests with control untreated net pieces did not exceed 10% while blood-feeding was > 50%. All 30 Interceptor® G2 nets passed the criteria for chlorfenapyr bioefficacy in the tunnel tests at baseline although this number fell to 19 at 12 months and was six and eight at 24 and 36 months, respectively.

**Table 7:** Results with Interceptor® G2 nets tested in tunnel tests against pyrethroid-resistant* Anopheles coluzzii* Akron strain mosquitoes at baseline and at 12-, 24- and 36-months post-distribution

Measures	Time points			
	Baseline	12 months	24 months	36 months
Number of ITNs tested	30	30	30	30
Number of ITN pieces tested	30	30	30	30
Number of mosquitoes exposed	3258	3308	3308	3218
Number of mosquitoes dead	3118	2715	1711	1938
Number of mosquitoes blood-fed	686	1028	1831	1512
% dead (95% CI)	95.7 (92.6–96.7)	82.1 (79.0–83.4)	51.7 (49.3–53.4)	60.2 (49.22–53.4)
% Blood-fed	21.1 (19.5–22.5)	31.1 (29.2–32.7)	55.4 (52.8–57.1)	47 (44.6–48.7)
% Blood-feeding inhibition	73.7	61.2	31.0	41.5
Number of ITNs passing WHO criteria	30/30	19/30	6/30	8/30

*CI* Confidence interval,* ITN* insecticide-treated net

#### Proportion of ITNs passing WHO criteria

**Fig. 6:**
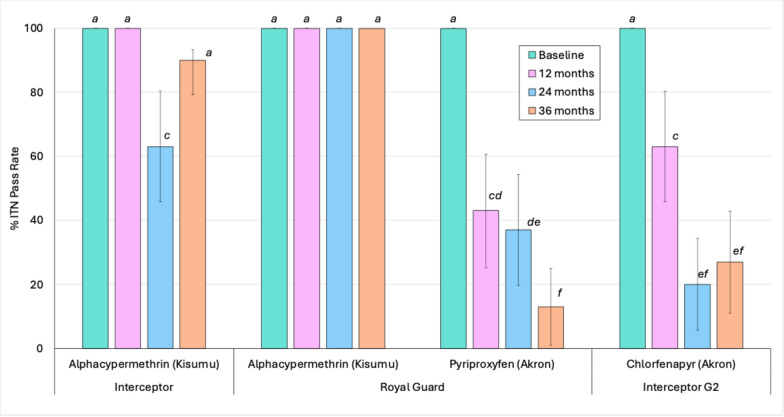
Proportion of ITNs that passed WHO efficacy criteria in the laboratory bioassays for each active ingredient tested. The Interceptor® ITN was tested against susceptible *Anopheles gambiae* Kisumu strain mosquitoes for alpha-cypermethrin bioefficacy in cone bioassays; the Royal Guard® ITN was tested against susceptible* An. gambiae* Kisumu strain mosquitoes for alpha-cypermethrin bioefficacy in cone bioassays and against blood-fed pyrethroid-resistant* Anopheles coluzzii* Akron strain mosquitoes for pyriproxyfen bioefficacy in cone bioassays; and Interceptor® G2 was tested against the pyrethroid-resistant* An. coluzzii* Akron strain in the tunnel tests. Each bar represents a sample size of 30 mosquito nets. Same lowercase letters above bars indicate that the pass rates at these time points are not significantly different (*p* > 0.05, Chi-square test). Baseline results were obtained with new unused nets. ITN, Insecticide-treated net

The percentages of ITNs of each type that passed efficacy criteria for each AI at each time point are presented in Fig. 6. The ITN pass rate for the bioefficacy of alpha-cypermethrin in Interceptor® was 100% at baseline and at 12 months although it declined to 65% at 24 months and later increased to 90% at 36 months. The Royal Guard® ITN showed a 100% ITN pass rate for the bioefficacy of alpha-cypermethrin throughout the study; in contrast, < 40% of Royal Guard® and Interceptor® G2 ITNs withdrawn at 24 months passed the WHO efficacy criteria for pyriproxyfen and chlorfenapyr, respectively. The proportion of ITNs passing the WHO bioefficacy criteria for pyriproxyfen in the Royal Guard® ITN and chlorfenapyr in the Interceptor® G2 ITN was generally similar at each time point (*p* > 0.05). These results showed a faster decline in bioefficacy of the non-pyrethroid insecticide compared to alpha-cypermethrin.

#### Changes in chemical content over time

**Fig. 7:**
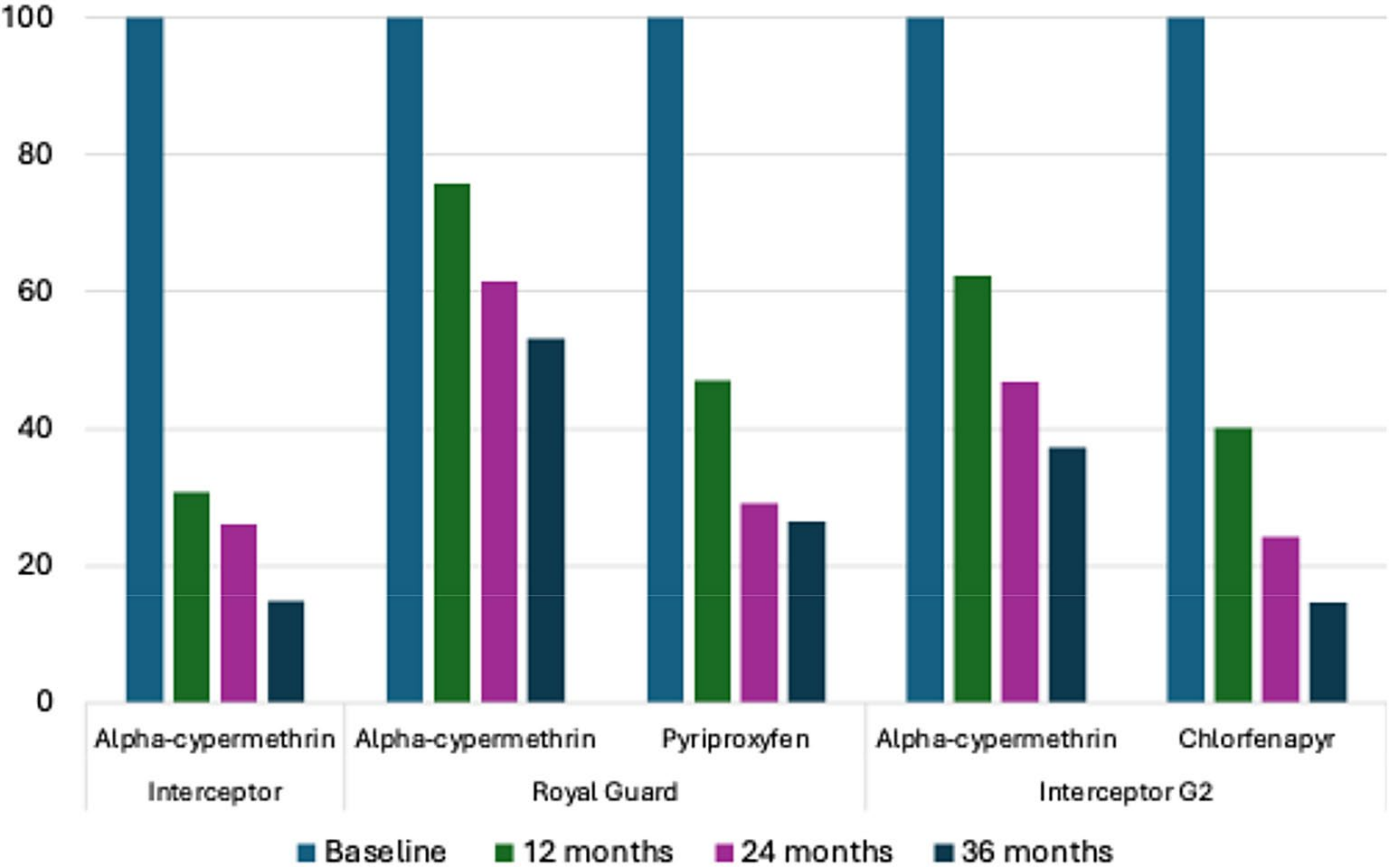
Percentage retention of active ingredient content of study nets over time

The mean content of the AI was within target expectations for each ITN type at baseline (Table 8). For both dual AI nets, greater amounts of alpha-cypermethrin were retained compared to the non-pyrethroid (Fig. 7). Over 50% of alpha-cypemethrin was still available in Royal Guard® nets at 36 months while the content of pyriproxyfen had fallen to 26% of the target. A similar trend was observed with Interceptor® G2 ITNs although the nets had lost > 85% of their chlorfenapyr content at 36 months.

**Table 8:** Chemical content (g/kg) of insecticide-treated nets withdrawn at 12-, 24- and 36-months post-distribution

Time point	Active ingredient parameters	ITN type				
		Interceptor®	Royal Guard®		Interceptor G2®	
		Alpha-cypermethrin	Alpha-cypermethrin	Pyriproxyfen	Alpha-cypermethrin	Chlorfenapyr
Baseline	Mean AI content (g/kg)	6.3	5.8	6.3	2.6	5
	95% CI	6.0–6.5	5.7–5.9	6.3–6.4	2.5–2.7	4.8–5.1
	% Relative SD	10.9	4.4	3	8.7	8.7
12 months	Mean AI content (g/kg)	1.9	4.4	3	1.6	2
	95% CI	1.6–2.3	4.1–4.7	2.6–3.4	1.4–1.8	1.5–2.5
	% Relative SD	44.4	18.5	37.9	34.3	71
	% Reduction in AI content	69.2	24.2	53	37.7	59.8
24 months	Mean AI content (g/kg)	1.6	3.6	1.8	1.2	1.2
	95% CI	1.1–2.2	3.2–3.9	1.5–2.2	1.0–1.4	0.8–1.7
	% Relative SD	94.3	24.7	44.4	47.8	101.4
	% Reduction in AI content	74	38.5	70.9	53.1	75.7
36 months	Mean AI content (g/kg)	0.9	3.1	1.7	1	0.7
	95% CI	0.6–1.2	2.7–3.5	1.3–2.1	0.8–1.2	0.4–1.1
	% Relative SD	90.6	33.7	66.3	60	125.3
	% Reduction in AI content	85.1	46.8	73.6	62.7	85.3

*AI* Active ingredient,* CI* confidence interval,* SD *standard deviation

## Discussion

In this study we evaluated the attrition, fabric integrity and insecticidal durability of two dual AI ITNs (Interceptor® G2 and Royal Guard®) compared to a WHO-prequalified alpha-cypermethrin net (Interceptor®) as part of cRCT performed in the Zou Department of Benin between 2020 and 2023. ITN durability studies are essential for planning their replacement and for making programmatic and procurement decisions.

In terms of ITN survivorship, the overall proportion of study nets available in households had fallen to 52% after 24 months, and only 15% of nets were still available after 36 months. Overall ITN median survival time was 2.0 years, which is substantially below the 3-year durability assumed in the Benin National Malaria Control Programme (NMCP) policy of triennial ITN campaign cycles. ITN attrition due to physical damage was generally low during the first 2 years (< 25%) but had increased substantially at the end point of the study (approx. 80%), indicating a significant loss in physical integrity of the study nets after 24 months of operational age. These findings align with those of the statistical inference study that reported poor ITN retention rates of < 2 years in most communities in Africa [4]. Nevertheless, an ITN coverage survey performed as part of the cRCT at 24 months showed that the study nets were mostly replaced by extra nets that householders still had in their possession from previous campaigns, thus maintaining an overall high ITN coverage of > 90% in the study area [9]. This finding highlights the need for strategies to optimise ITN quantification and allocation to ensure equitable access across target populations [3].

Our comparison of ITN survivorship between the three ITN types demonstrated that the Interceptor® and Interceptor® G2 ITNs had a higher median survival time (2.3 and 2.1 years, respectively) than the Royal Guard® ITN (1.6 years). This result was further supported by the lower proportion of Royal Guard® nets hanging over sleeping places compared to Interceptor® and Interceptor® G2 and their increased replacement with other non-study nets. These findings can be attributed to differences in fabric type between the study nets; Interceptor® and Interceptor® G2 ITNs are made of polyester fabric while Royal Guard® is made of polyethylene fabric. Indeed, the qualitative survey performed 24-months post-distribution showed that householders overwhelmingly preferred ITNs made of polyester fabric due to their softer texture compared to ITNs made from polyethylene fabric, and this preference may have driven the higher replacement of Royal Guard® nets, mostly with spare polyester nets (mainly PermaNet® 2.0) from the previous ITN campaigns. This may also have contributed to the lack of an improved epidemiological impact with Royal Guard® relative to Interceptor® in the cRCT [9]. ITN users’ low preference for the polyethylene ITN fabric texture has been demonstrated in multiple studies in Africa [25]. While an earlier study evaluating the durability of a polyethylene net (Olyset® Plus; Sumitomo Chemical, Tokyo, Japan) distributed in Benin in 2010 reported a median survival time of just 2 years [26], more recent durability studies with polyester ITNs in Benin have shown longer median survival times of 2.8 years [27]. It has been argued that householders tend to use the net types that are available to them and that ITN use at the population level may not be affected by the user’s fabric texture preference [28], but the findings from our study indicate that householders in Benin would be inclined to discard polyethylene nets more quickly than they would polyester nets. This finding supports the Benin NMCP’s policy of excluding polyethylene nets from their mass ITN campaigns. Given that the polyethylene fabric texture is more suitable for the application of some non-pyrethroid compounds on ITNs, strategies to improve the softness of polyethylene texture used for ITNs may help improve their acceptability and use by householders.

The laboratory bioassays showed better retention of the bioefficacy of the pyrethroid component in the dual AI ITNs over 3 years of operational use compared to the non-pyrethroid component. The proportion of ITNs passing predetermined efficacy criteria in the bioassays had fallen to < 40% at 24 months of operational age for pyriproxyfen in the Royal Guard® ITN and chlorfenapyr in the Interceptor® G2 ITN, while 100% of Royal Guard® nets passed efficacy criteria for the pyrethroid component throughout the study. This result can be explained by the higher loss rate of the non-pyrethroid active ingredients from the dual AI nets relative to the pyrethroid AI. Studies have also shown a significantly higher loss of piperonyl butoxide (PBO) (> 80%) compared to pyrethroids on pyrethroid-PBO ITNs as they age under operational conditions [29–31]. These findings suggest that pyrethroids are generally more durable on ITNs than non-pyrethroid compounds used on existing dual AI nets. This further highlights the need for more innovative technologies to improve the insecticidal durability of non-pyrethroid AIs on mosquito nets.

As new non-pyrethroid AIs are applied to ITNs, the methods and strains used for monitoring their bioefficacy under operational conditions must align with their mode of action. In this study, we modified existing WHO bioassays to allow assessment of the delayed toxic effects of chlorfenapyr in the Interceptor® G2 ITN using tunnel tests and the sterilising effects of pyriproxyfen in the Royal Guard® ITN by dissecting the ovaries of exposed blood-fed females. The bioassays provided interpretable results that aligned with changes in the content of the non-pyrethroid AIs over time. Bioassays that did not meet predefined validity criteria, such as cut-offs for mortality, blood-feeding or fertility, among others, in control unexposed mosquitoes were very rare. The susceptibility data also showed that the pyrethroid-resistant *An. coluzzii* Akron strain mosquitoes used for bioefficacy testing of the non-pyrethroids maintained its resistance status throughout the study. This suggests that the methods used for monitoring the bioefficacy of both non-pyrethroid AIs over time were appropriate and could therefore contribute to the development of new WHO guidelines for investigating the bioefficacy of WHO-prequalified dual AI ITNs under long-term community use.

While the laboratory bioassays demonstrated how the bioefficacy of each ITN type changed over time, they did not provide a direct comparison between the ITN types given that different methods and mosquito strains had to be used to monitor changes in bioefficacy of each AI over time. Experimental hut trials were thus performed in parallel against wild free-flying pyrethroid-resistant malaria vectors in the Cove hut station, with study nets withdrawn from households at 12, 24 and 36 months of operational age (unpublished data). The hut trial results showed the superiority of Interceptor® G2 nets over other dual AI ITN types although the improved impact relative to Interceptor® had declined significantly with 24- and 36-months nets. This result is supported by the substantial reduction in the proportion of Interceptor® G2 nets passing criteria for chlorfenapyr bioefficacy in the laboratory bioassays after 24 months in the present study relative to Interceptor® nets (< 30% vs 63–90%, respectively). The reduced bioefficacy and chemical content of chlorfenapyr in Interceptor® G2 ITNs may therefore partly explain the loss of its improved epidemiological impact relative to that of the Interceptor® ITN in the third year of the cRCT [32]. Given the dependence of most malaria-endemic countries on triennial ITN campaigns for malaria prevention and control, innovative strategies and cost-effective interventions that can be deployed to improve vector control in the third-year post-ITN distribution are needed.

## Conclusions

This study demonstrated a median ITN survival time of 2.1 years for the Interceptor® G2 ITN and 1.6 years for the Royal Guard® ITN in communities in Benin. Among the three ITNs tested, Royal Guard® nets were less used and discarded more quickly by householders partly due to householders’ low preference for polyethylene nets relative to polyester nets. The insecticidal efficacy and content of the pyrethroid AI was more durable relative to that of the non-pyrethroid AIs in the Interceptor® G2 and Royal Guard® ITNs. All Royal Guard® nets passed the bioefficacy criteria for the pyrethroid component at 36 months while only 14% passed the bioefficacy criteria for pyriproxyfen at the same time point. For Interceptor® G2, only 25% of nets passed the bioefficacy criteria for chlorfenapyr at 36 months. The content of the non-pyrethroid insecticides at 36 months had fallen by 85% for chlorfenapyr in Interceptor® G2 and by 73% for pyriproxyfen in Royal Guard® as opposed to 65% and 47% for the alpha-cypermethrin content, respectively. The results of this study corroborate the findings from the cRCT.

## Supplementary Information

Additional file 1. Table S1.

Additional file 2.

## Data Availability

The datasets used and/or analysed during the present study are available from the corresponding author upon reasonable request.

## Abbreviations

AI: Active ingredient
cRCT: Cluster randomised controlled trial
CREC: Centre de Recherche Entomologique de Cotonou
ITNs: Insecticide-treated nets
LLINs: Long-lasting insecticidal nets
LSHTM: London School of Hygiene & Tropical Medicine
NMCP: National Malaria Control Programme
PBO: Piperonyl butoxide
pHI: Proportionate hole index
SSA: Sub-Saharan Africa
