# Supplementary material for: The attrition, physical and insecticidal durability of two dual active ingredient nets (Interceptor® G2 and Royal Guard®) in Benin, West Africa: results from a durability study embedded in a cluster randomised controlled trial
Source: Parasit Vectors. 2024 Oct 7;17:420. doi: 10.1186/s13071-024-06504-1 (PMC11459981; doi:10.1186/s13071-024-06504-1)
Supplement: Supplementary file 1 — Additional file 1. Table S1. [file 13071_2024_6504_MOESM1_ESM.docx]

**The physical and insecticidal durability of two dual active ingredient nets (Interceptor® G2 and Royal Guard®) in Benin, West Africa; results for a durability study embedded in a cluster randomised control trial**

**Authors:** Corine Ngufor^1, 2, 3*^, Josias Fagbohoun^2,3^, Augustin Fongnikin^2,3^, Thomas Syme^1,2,3^, Juniace Ahoga^2,3^, Idelphonse Ahogni^2,3^, Manfred Accrombessi^1,2^, Natacha Protopopoff^1^, Jackie Cook^1^, Edouard Dangbenon^2^, Arthur Sovi^1,2^, Marie Baes^4^, Olivier Pigeon^4^, Damien Todjinou^2,3^, Renaud Govoetchan^1,2,3^, Germain Gil Padonou^2^, Martin Akogbeto^2^

**Supplementary information**

Table S1: Summary of laboratory bioassay methodology and outcome measures for assessing the insecticidal durability of Interceptor® G2 and Royal Guard® in Benin compared to Interceptor®

| **ITN type** | **Brand name** | **Active ingredient** | **Mosquito strain** | **Mosquito age and status (N)** | **Primary test method**  **(exposure time)** | **Key outcome measures** | **ITN efficacy criteria** | **Remarks/additional tests** |
| --- | --- | --- | --- | --- | --- | --- | --- | --- |
| **Pyrethroid-only** | Interceptor | alpha-cypermethrin | Susceptible *An. gambiae* ss Kisumu | unfed, 3-5 days old  (40-50/net) | cone bioassays  (3 minutes) | Knockdown, 24h Mortality | knock-down ≥ 95% or mortality ≥ 80% | Tunnels for failed nets |
| **Pyrethroid-chlorfenapyr** | Interceptor® G2***^a^*** | alpha-cypermethrin | not tested | not tested | not tested | not tested | not tested | not tested due to lower dose of pyrethroid |
|  |  | chlorfenapyr | Pyrethroid-resistant *An. coluzzii* Akron | unfed, 5-8 days old  (100/net) | Tunnel tests  (overnight) | 72h Mortality and Blood-feeding inhibition | Mortality ≥ 80% or Blood-feeding inhibition ≥ 80% | 1 net piece per ITN |
| **Pyrethroid- pyriproxyfen** | Royal Guard | alpha-cypermethrin | Susceptible *An. gambiae* ss Kisumu | unfed, 5-8 days old  (40-50/net) | cone bioassays  (3 minutes) | Knockdown, Mortality | knock-down ≥ 95% or mortality ≥ 80% | Tunnels for failed nets |
|  |  | pyriproxyfen | Pyrethroid-resistant *An. coluzzii* Akron | blood-fed, 5-8 days old  (80-100/net) | cone bioassays  (3 minutes) plus ovary dissection after 72h | % reduction in fertility relative to control***^b^*** | ≥ 50% reduction in fertility***^c^*** | >30% fertility required in control |

^a^For Interceptor® G2, given the low dose of alpha-cypermethrin, only chlorfenapyr bioefficacy was assessed

^b^Mosquitoes presenting with Christopher stage V ovaries, were considered fertile.

^c^A cut-off of 50% was applied for bioefficacy of pyriproxyfen based on preliminary studies with Royal Guard® nets washed 20 times.
