# Supplementary material for: The attrition, physical and insecticidal durability of two dual active ingredient nets (Interceptor® G2 and Royal Guard®) in Benin, West Africa: results from a durability study embedded in a cluster randomised controlled trial
Source: Parasit Vectors. 2024 Oct 7;17:420. doi: 10.1186/s13071-024-06504-1 (PMC11459981; doi:10.1186/s13071-024-06504-1)
Supplement: Supplementary file 2 — Additional file 2. [file 13071_2024_6504_MOESM2_ESM.docx]

**Supplementary information**

**The attrition, physical and insecticidal durability of two dual active ingredient nets (Interceptor® G2 and Royal Guard®) in Benin, West Africa; results for a durability study embedded in a cluster randomised controlled trial**

**Authors:** Corine Ngufor^1, 2, 3*^, Josias Fagbohoun^2,3^, Augustin Fongnikin^2,3^, Juniace Ahoga^2,3^, Thomas Syme^1,2,3^, Idelphonse Ahogni^2,3^, Manfred Accrombessi^1,2^, Natacha Protopopoff^1^, Jackie Cook^1^, Edouard Dangbenon^2^, Arthur Sovi^1,2^, Marie Baes^4^, Olivier Pigeon^4^, Damien Todjinou^2,3^, Renaud Govoetchan^1,2,3^, Germain Gil Padonou^2^, Martin Akogbeto^2^


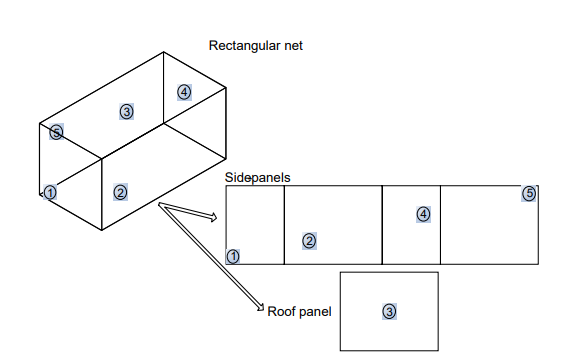


Figure S1: Sampling pattern of net pieces from study nets[1]. Position 1 is only sampled at baseline.

1. WHO. Guidelines for monitoring the durability of long-lasting insecticidal mosquito nets under operational conditions. Geneva, Switzerland: World Health Organization. 2011.
